# The burden of COVID-19 infection on medical doctors in the first year of the pandemic in Ghana

**DOI:** 10.4314/gmj.v56i2.3

**Published:** 2022-06

**Authors:** Titus K Beyuo, Emma R Lawrence, Richard Selormey, Samuel E Fosu, Frank K Ankobea

**Affiliations:** 1 Department of Obstetrics and Gynaecology, University of Ghana Medical School, Accra, Ghana; 2 Department of Obstetrics and Gynaecology, University of Michigan, USA; 3 St. Elizabeth Catholic Hospital, Hwidiem, Ahafo Region, Ghana; 4 iMentor Ghana; 5 School of Medical Sciences, Kwame Nkrumah University of Science and Technology

**Keywords:** COVID-19, Ghana, workplace infection, occupational infection

## Abstract

**Objective:**

To quantify and describe the burden of COVID-19 infection amongst doctors in Ghana

**Design:**

A quantitative and qualitative analysis of cross-sectional data was performed.

**Setting:**

All 16 regions in Ghana.

**Participants:**

Participants were medical doctors diagnosed with COVID-19 between March 2020 and March 2021.

**Main outcome measures:**

Data sources were Ghana Medical Association and Ministry of Health records. Demographics and workplace data included age, gender, the rank of the doctor, and location and type of current facility. Characteristics of the COVID-19 infection included the likely source, clinical and recovery status, and place of management. Doctors reported their desire for a general checkup and psychological support and described the challenges encountered.

**Results:**

The prevalence of COVID-19 infection was 88.9 cases per 1000 doctor-population. Of 544 infected doctors, 59.2% were stable but symptomatic, and 1.7% were in critical condition, with a case fatality rate of 1.7%. Overall, 31.6% had recovered from their COVID-19 infection, and the majority (82.4%) were managed at home in self-isolation. Compared to medical officers, house officers (OR 1.36, p=0.03), senior house officers (OR 7.60, p<0.001), and consultants (OR 2.94, p=0.001) were more likely to have a COVID-19 infection. Desire for support was varied, with 13.0% desiring someone to check on them and 9.7% desiring psychological support. The majority (75.3%) reported facing a challenge, including difficulty obtaining needed vitamins and medications, and accessing daily necessities like groceries.

**Conclusions:**

In Ghana, COVID-19 infections greatly burden medical doctors.

**Funding:**

None declared

## Introduction

The severe acute respiratory syndrome coronavirus 2 (SARS-CoV-2), which causes COVID-19, has caused an enduring pandemic with significant impact worldwide. On January 12, 2020, the World Health Organization (WHO) attributed a cluster of respiratory illnesses in Wuhan, China to a novel coronavirus, COVID-19.^1^ Although a large proportion of infected individuals were asymptomatic, many experienced mild respiratory symptoms like fever, cough, and shortness of breath. Some developed acute respiratory distress and required hospitalisation and intubation.^2^ In infected individuals, higher rates of complications and death have been seen in the elderly and people with underlying cardiac and respiratory disease.^3^

As of March 2021, there are more than 128 million cases of COVID-19 and 2.8 million deaths globally.^4^

The first cases of COVID-19 in Ghana were documented on March 12, 2020, by the Noguchi Memorial Institute for Medical Research laboratory, in individuals who had returned from international travel.^5^ Public health measures taken to contain the spread of COVID-19 in Ghana included a ban on all public gatherings and closure of schools and religious centres on March 16, an international travel ban on entry for travellers coming from a country with more than 200 COVID-19 cases on March 17, quarantine of international travellers on March 22, and a partial lockdown of major urban centers on March 30.^6^,^7^

The lockdown was lifted on April 20, 2020. However, continued public health measures remained in place, including a mandate to wear facemasks in public areas, a ban on large social gatherings, and public health campaigns on hand hygiene. Initially, testing for COVID-19 was performed at the Noguchi Memorial Institute for Medical Research, Accra, and Kumasi Centre for Collaborative Research in Tropical Medicine. Testing sites have now been increased to include multiple sites throughout the country. 6 One year after Ghana's first cases, as of March 12, 2021, the Ghana Health Service reported 3,701 active cases, a history of 88,228 confirmed cases in Ghana, and 698 deaths.^8^

Across the world, health systems have struggled to pro-vide care to patients during the COVID-19 pandemic while ensuring personal protective equipment (PPE) and protocols to protect their healthcare workers.^9^,^11^ COVID-19 disproportionately impacts healthcare providers on the front line of treating patients with COVID-19. Even after adjusting for differences in testing frequency, healthcare workers were more than three times more likely to be infected by COVID-19 than the general public.^12^ The highest risk of infection was seen in healthcare workers using inadequate PPE. However, even healthcare workers who reported using adequate PPE had an increased susceptibility to infection.^12^ Healthcare workers in low-resource settings like Ghana faced additional challenges, including limitations in the availability of PPE and access to rapid testing. The World Health Organization (WHO) estimates that Ghana has just 1.8 medical doctors and 42 nurses and midwives per 10,000 population. Thus, the healthcare workforce is overburdened. 13 In health centers that are understaffed at baseline, prolonged absence of doctors due to infection can be stressful for the healthcare system, the remaining doctors and nurses, and the infected doctors themselves.

In addition to the public health prevention protocols, vaccination was considered a critical step for nations to combat this pandemic. A global search for safe and effective vaccines yielded results with many high-resource countries carrying out national vaccination initiatives, with healthcare workers prioritising receiving vaccines.^14^–^15^ Due to inequitable distribution of vaccines, most low to middle-income countries (LMICs) had delayed and inadequate supply of vaccines.^16^ Ghana started its vaccination program in March 2021, approximately a year after reporting its first case of COVID-19.^17^

This study aimed to quantify the burden of COVID-19 on doctors in Ghana during the first year of the outbreak and before a full-scale rollout of vaccination, describe the distribution of infected doctors by demographic and workplace factors, and explore self-reported challenges. A better understanding of the profile of doctors infected with COVID-19, and their characteristics and challenges, will inform hospital policies to best support these vulnerable frontline workers in this COVID-19 pandemic and in future pandemics of similar nature.

## Methods

This study is a quantitative and qualitative analysis of cross-sectional data. Participants are doctors in Ghana who were diagnosed with COVID-19 between March 2020 and March 2021. Ethical approval was granted by the Ghana Health Service Ethics Review Committee (GHS-ERC: 020/07/21). Participant consent was not obtained given that this was an analysis of already collected de-identified data.

Data on the total number of doctors employed by the government of Ghana was obtained from the Ministry of Health (MOH) payroll records. The data were categorised by the grade of the doctor. Data on all doctors in Ghana confirmed COVID-19 infection were obtained from the Ghana Medical Association (GMA) records. The data consisted of de-identified individual-level data. Demographic data included age and gender. Workplace data included the rank of the doctor, current healthcare facility, region of the current facility, and type of facility. Data on characteristics of the COVID-19 infection included the likely source of the COVID-19 infection, current clinical status, recovery status, presence of comorbid medical conditions, and place of management. Recovered status was defined as two consecutive negative COVID-19 tests and full resolution of symptoms. Data on desired support for COVID-19 infected doctors included responses to two yes/no questions on the desire for a general checkup and desire for psychological support. Aside from age which was collected as a continuous variable, and then categorised by decade for analysis, all other variables were collected as categorical variables. Finally, infected doctors were asked about the presence and nature of challenges they encountered as part of their COVID-19 experience. This data was collected as a short response.

### Data Analysis/Calculations

Data were organised and analysed using Microsoft Excel and R respectively. Data on demographics, workplace factors, and characteristics of the COVID-19 infection were described using means and standard deviations for continuous variables and frequencies and proportions for categorical variables.

The prevalence of COVID-19 among doctors was calculated using the number of infected doctors as the numerator and the number of total doctors working in Ghana as the denominator. Case fatality rate was calculated using the number of deaths among infected doctors as the numerator and the number of total infected doctors as the denominator; the ratio was then multiplied by 100 to calculate the rate. Odds ratios were used to calculate the comparative odds of COVID-19 infection by rank, among doctors employed by the government of Ghana. To calculate the odds ratios, the MOH payroll record was used to determine the total number of government-employed doctors by rank. The data from the GMA on infected doctors included government-employed doctors as well as private practitioners and other categories not on the government payroll. To determine the percentage of doctors infected by rank and all subsequent analyses, all doctors not employed by the government, including doctors working at private hospitals and non-governmental organisations (NGOs) were excluded from the GMA data.

Finally, a qualitative analysis of short answer responses to the question “what were the challenges you faced or still face during management?” was conducted, using a standard Attride-Stirling approach.^18^ All narrative short answer comments were reviewed. Through incremental and iterative coding of the comments, we arrived at four-teen keyword phrases for challenges faced. After our list of generated themes achieved stability, the coding process was repeated for all responses using our final comprehensive list. Each comment could be coded with up to 5 different keyword phrases, to capture multi-themed comments.

## Results

From March 2020 to March 2021, 544 doctors were infected with COVID-19 in Ghana. There were 6,117 registered doctors in Ghana during this period, resulting in an estimated prevalence of 88.93 cases per 1000 population (8.9%). Doctors with COVID-19 infections had a mean age of 33.4 years with most infected doctors between ages 30 and 40 years (Table 1). Of all infected doctors, 58.8% (n=320) were male and 41.2% (n=224) were female. The most common rank of infected doctors was medical officer (23.5%, n=128), followed by house officer (17.6%, n=96). There was representation from a wide range of regions across Ghana, with most doctors from the Greater Accra (42.8%, n=232) and Ashanti (32.3%, n=175) regions.

**Table 1:** Characteristics of doctors infected with COVID-19 in Ghana during the first 12 months of the pandemic

Variable	Frequency (proportion)
Age (mean +/- sd)	33.4 +/- 7.6
Age category	
Less than 20	0 (0.0%)
20 to <30	119 (33.4%)
30 to <40	197 (52.7%)
40 to <50	36 (9.6%)
50 to <60	11 (2.9%)
60 to <70	3 (0.8%)
70 and older	2 (0.5%)
Gender (n, %)	
Male	320 (58.8%)
Female	224 (41.2%)
Grade/Rank (n, %)	
Consultant	17 (3.1%)
Senior Specialist	13 (2.4%)
Specialist	58 (10.7%)
Senior resident	31 (5.7%)
Resident	80 (14.7%)
Chief medical officer	1 (0.2%)
Deputy chief medical officer	1 (0.2%)
Principal medical officer	7 (1.3%)
Senior medical officer	45 (8.3%)
Medical officer	128 (23.5%)
Senior house officer	63 (11.6%)
House officer	96 (17.6%)
Others	2 (0.4%)
Region (n, %)	
Ashanti	175 (32.3%)
Brong-Ahafo (Bono, Bono East and Ahafo Regions)	12 (2.2%)
Central	23 (4.2%)
Eastern	17 (3.1%)
Greater Accra	232 (42.8%)
Northern (Northern, Savana, North East Regions)	46 (8.5%)
Upper East	2 (0.4%)
Upper West	1 (0.2%)
Volta (Volta and Oti Regions)	16 (3.0%)
Western (Western and Western North Regions)	18 (3.3%)
Type of Institution (n, %)	
Christian Health Association of Ghana (CHAG)	56 (10.3%)
Ghana Health Service (GHS)	126 (23.2%)
Ministry of Health (MOH)	3 (0.6%)
Non-Governmental Organization (NGO)	2 (0.4%)
Private hospital	17 (3.1%)
Quasi government hospital	57 (10.5%)
Teaching hospital	281 (51.7%)
Others	2 (0.4%)

**Figure 1:**
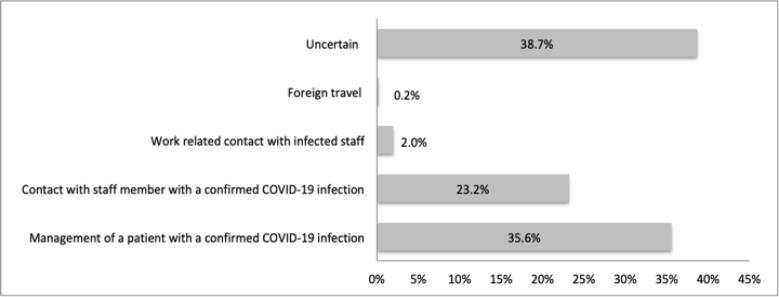
Self-reported likely source of personal COVID-19 infection, among doctors in Ghana

**Figure 2:**
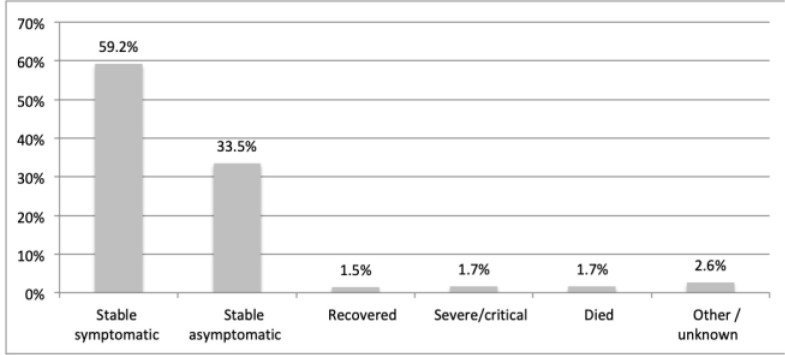
Current clinical status among doctors in Ghana infected with COVID-19

Regarding clinical characteristics of the COVID-19 infection, 38.7% (n=210) reported their likely source of infection was uncertain, 35.6% (n=193) from the management of a patient with confirmed COVID-19 infection, and 23.2% (n=126) from contact with a staff member with a COVID-19 infection (Figure 1). The majority of infected doctors were stable but symptomatic (59.2%, n=133) and 1.7% (n=9) were in severe/critical condition (Figure 2).

Overall, 31.6% (n=172) report that they had recovered from their COVID-19 infection (Table 2).

**Table 2:** Characteristics of COVID-19 Infections, Among Infected Doctors in Ghana

Variable	Frequency (proportion)
Recovery status (n, %)	
Recovered	172 (31.6%)
Not recovered	314 (57.7%)
Unknown	58 (10.7%)
Comorbidities (n, %)	
Liver/gastrointestinal disease	2 (0.4%)
Pulmonary disease, including asthma	30 (5.5%)
Cardiovascular disease, including hypertension	27 (5.0%)
Diabetes	5 (0.9%)
Allergies	3 (0.6%)
Current pregnancy	2 (0.4%)
Sickle cell disease	2 (0.4%)
Other	5 (0.9%)
Place of management (n, %)	
Home – self isolation	448 (82.4%)
Public isolation centre	9 (1.7%)
Partly self-isolation and partly facility based isolation	17 (3.1%)
Employer provided isolation	7 (1.3%)
Treatment centre	25 (4.6%)
Intensive care unit (ICU)	9 (1.7%)
Other	29 (5.3%)

The majority of doctors with COVID-19 (82.4%, n=448) were managed at home in self-isolation. Compared to medical officers, the odds that house officers had a COVID-19 infection was 1.36 times higher (OR 1.36, p=0.03), and the odds that senior house officers had a COVID-19 infection were 7.60 times higher (OR 7.60, p<0.001), and the odds that consultants had a COVID-19 infection was 2.94 higher (OR 2.94, p<0.001) (Table 3).

**Table 3:** C30OVID-19 infection by rank, among govern-ment-employed doctors

Grade / Rank	Total employed	Confirmed with COVID	No disease	Odds ratio	p-value
Medical officer	1626	119	1507	Ref	Ref
House officer	991	96	895	1.36	**0.03**
Senior house officer	168	63	105	7.60	**< 0.001**
Senior Medical Officer	771	41	730	0.71	0.07
Specialist	831	57	774	0.93	0.70
Senior Specialist	236	13	223	0.73	0.31
Consultant	85	16	69	2.94	**0.001**

In total, nine Ghanaian doctors experienced a COVID-19 related mortality, resulting in a case fatality rate of 1.7%. Of the nine doctors who died, 4 (44.4%) were consult-ants, 2 (22.2%) were specialists and 3 (33.3%) were general practitioners in private practice. These doctors practised at different hospitals across Ghana, with some in senior management positions, with specialties including internal medicine (1), paediatrics (2), surgery (1), orthopaedics (1), obstetrics and gynaecology (1) and general practice (3). Six (66.7%) of these doctors were 60 years or older.

Among doctors, the desire for support varied, with 13.0% (n = 18 of 134 who responded to this question) desiring someone to check on them and 9.7% (n = 13 of 134 who responded to it) desiring a psychological consult facilitated by the GMA. Of the study population, 198 responded to the short answer questions about challenges faced (Table 4). Of those respondents, 49 (24.7%) reported that they did not face any challenges, and 149 (75.3%) replied with a short description of one or more challenges. The most prevalent theme that emerged was difficulty in obtaining needed vitamins and medications due to availability, accessibility, and cost issues. The second most common theme was difficulty accessing daily necessities like groceries in isolation, especially among individuals who lived alone. Issues with testing were commonly reported, with delays in test results as the most prevalent testing challenge. Many respondents also cited a lack of support, communication, and follow-up from the workplace and COVID-19 care teams.

Table 4Challenges experienced by COVID-19 infected doctors

**Table 4a:** Presence of Challenges

What were the challenges you faced or still face during management?	Total n = 198 responses n(%)
No challenges	49 (24.7%)
Faced a challenge	149 (75.3%)

**Table 4b:** Challenge Themes

Challenge theme	Specific challenge description	Proportion (frequency) with the challenge (n=149) *
**Mental** **health**	Feeling of isolation / loneliness	5.4% (8/149)
	Anxiety or depression	2.0% (3/149)
	Stigmatization	3.4% (5/149)
**Testing**	Delays in test results	17.4% (26/149)
	Difficulty accessing testing	6.7% (10/149)
	High cost of testing; burden to pay for testing themselves	4.0% (6/149)
	Inadequate contract tracing; difficulty getting family members tested	8.7% (13/149)
**Family**	Concern about infecting family difficult to isolate from family	4.0% (6/149)
**Daily needs**	Difficulty safely accessing groceries	18.1% (27/149)
**Medical** **care**	Difficulty assessing medications; burden to pay for medications themselves	28.9% (43/149)
	Experiencing clinical symptoms	9.4% (14/149)
	Lack of care management protocols and follow-up	7.4% (11/149)
**Support**	Lack of support from the workplace	12.1% (18/149)
	Lack of psychological support	4.7% (7/149)

* Percentages reflect the number of short answer re-sponses that incorporate each theme. Each response could be coded with up to 5 themes. Thus, percentages add to greater than 100%.

## Discussion

In the first year of the COVID-19 pandemic, 544 doctors were infected with COVID-19 in Ghana. Our study demonstrates a large burden of the pandemic on medical doctors in the country. The majority of infected doctors were stable and symptomatic, but 1.7% were in critical condition, and 1.7% died. Compared to medical officers, house officers, senior house officers, and consultants were more likely to have a COVID-19 infection. This may be explained by longer hours of patient contact practised by house officers, as well as older age and potentially higher rates of comorbid conditions among consultants. Most doctors reported facing a challenge, including issues with mental health, medication access, testing delays, and desire for additional support.

Globally, healthcare providers have been at a substantial risk of being infected with COVID-19.^19^ COVID-19 spreads through contact with respiratory droplets, bodily fluids, or contaminated surfaces, all of which are common in hospital settings.^20^ The highest risk clinical situations for contraction of COVID-19 include aerosolising procedures like intubation, nebulisation and noninvasive positive pressure ventilation.^21^ Importantly, asymptomatic patients can also transmit the SARS-CoV-2 virus before clinical suspicion or before testing results are available. In a 2020 survey of 472 healthcare workers across Ghana, only 27.8% of healthcare workers felt prepared to deal with COVID-19 in the workplace. Factors associated with higher perceived preparedness were having undergone training, access to adequate PPE, presence of an isolation ward at their health center, protocols for screening, and good communication from management.^9^

In this study, 35.0% of doctors reported their likely source of infection to be through an infected patient. Risks for providers becoming infected with COVID-19 include prolonged work hours, prolonged exposure to infected patients, inadequate hand-washing practices, and inadequate PPE. PPE was inadequate globally, especially at the start of the pandemic, and even more so in low-resource settings. N95 respirators, which provide a higher level of protection for at-risk providers, were especially costly. Providing adequate PPE is essential to reduce the risk of transmission of COVID-19 to healthcare workers. PPE includes not only facemasks and N95 respirators as appropriate but also goggles, protective gowns, and gloves.^20^ In addition, protocols on using, re-using, and storing PPE are important. As knowledge about COVID-19 continues to evolve, regular training and re-training on PPE protocols will allow healthcare workers to use evidence-based protection.

In addition to personal risk, infected healthcare providers are a vector for in-hospital spread to other healthcare providers and patients and may also infect their family members.^19^ This high risk for infection and high concern for infectivity may result in psychological distress among healthcare providers. In the literature, psychological distress secondary to COVID-19 is greatest among providers who are younger, more junior and have dependent children.^19^ A survey of Ghanaians demonstrated lower mental health scores resulting from social isolation during COVID-19.^22^

The WHO has asserted that protecting healthcare workers from COVID-19 should be a priority.^23^ The WHO has developed a risk assessment tool to categorise an exposure of a healthcare worker to a patient with COVID-19. Using this risk assessment tool, a survey among 408 frontline workers in designated COVID-19 treatment centers in Ghana found that 80.4% of healthcare workers had occupational exposure to COVID-19, with 14.0% classified as high-risk exposures.^24^ According to the WHO, providers at low risk of infection from their nosocomial exposure should self-monitor temperature and respiratory symptoms daily for 14 days after the last day of exposure to a COVID-19 patient, however, it is appropriate to continue work with routine PPE and handwashing. Providers at high risk of infection should stop all health care interactions with patients for a period of 14 days after the last day of exposure to a confirmed COVID-19 patient. Providers should also be tested for COVID-19 and should quarantine for 14 days. Importantly, in the event of infection or need for quarantine, the WHO also recommends that health care facilities provide psychosocial support and compensation during quarantine or illness.^23^

Strengths of this study include both a quantitative and qualitative approach used to evaluate an important population that is uniquely affected by the COVID-19 pandemic. Since the data is self-reported by doctors, we expect clinical information like recovery status, current condition, and medical comorbidities to be accurate. Our study is limited by the self-reported nature of our data, which could introduce errors or bias.

Since data was originally provided to the GMA along with identifying information, doctors may have been hesitant to discuss personal challenges. Thus, the true rate of challenges may be higher than reported. Data from the MOH on total doctors in Ghana included a breakdown by the rank of doctor but did not include information on other factors like gender or location. Thus, we were unable to calculate the odds ratios of infection across variables aside from rank. This study was conducted during the first year of the COVID-19 pandemic when protocols were limited, PPE was more scarce, and vaccination was unavailable. Thus, study findings may have limited generalizability to current settings in which healthcare providers have access to vaccination. Since this study was an analysis of previously collected data, it is limited by the inability to ask participants follow-up or clarifying questions. Additional studies are needed to provide an in-depth qualitative exploration of challenges faced by doctors. Additional research is also needed to understand the long-term impact of COVID-19 on doctors in Ghana and the characteristics of COVID-19 infection among nurses and other healthcare practitioners in Ghana.

## Conclusion

The COVID-19 pandemic had a large burden on medical doctors in Ghana; 544 doctors were infected during the first year of the pandemic. The odds of being infected differed based on the rank of the doctor, implying the need for tailor-made protective measures based on rank and job description. Allocation of limited PPE, additional infection prevention training, and COVID-19 testing could be considered in higher-risk groups.
